# Comparison of Structural Architecture of HCV NS3 Genotype 1 versus Pakistani Genotype 3a

**DOI:** 10.1155/2014/749254

**Published:** 2014-10-21

**Authors:** Kaneez Fatima, Esam Azhar, Shilu Mathew, Ghazi Damanhouri, Ishtiaq Qadri

**Affiliations:** ^1^IQ Institute of Infection and Immunity, P.O. Box 54000, Lahore, Punjab, Pakistan; ^2^King Fahd Medical Research Center, King Abdul Aziz University, P.O. Box 80216, Jeddah 21589, Saudi Arabia; ^3^Center of Excellence in Genomic Medicine, King Abdul Aziz University, P.O. Box 80216, Jeddah 21589, Saudi Arabia

## Abstract

This study described the structural characterization of Pakistani HCV NS3 GT3a in parallel with genotypes 1a and 1b NS3. We investigated the role of amino acids and their interaction patterns in different HCV genotypes by crystallographic modeling. Different softwares were used to study the interaction pattern, for example, CLCBIO sequence viewer, MODELLER, NMRCLUST, ERRAT score, and MODELLER. Sixty models were produced and clustered into groups and the best model of PK-NCVI/Pk3a NS3 was selected and studied further to check the variability with other HCV NS3 genotypes. This study will help in future to understand the structural architecture of HCV genome variability and to further define the conserved targets for antiviral agents.

## 1. Background

The hepatitis C virus (HCV) is a positive-sense, approximately 9.6 kilo base (kb) single-stranded RNA (ssRNA) which is translated into a polypeptide of over 3,000 amino acids long from a single open reading frame. HCV belongs to genus* Hepacivirus* of* Flaviviridae* family [1] and sole member of its family. Both the host and viral proteases process the HCV polyprotein into four structural proteins (core, E1, E2, and p7) and six nonstructural (NS) proteins (NS2, NS3, NS4A, NS4B, NS5A, and NS5B) [2].

HCV existed as quasispecies due to its better evolutionarily stable strategy. To date six genotypes [1–7] and more than 100 subtypes have been described [3]. These subtypes can exist as separate or in combination in an individual [4]. In Pakistan more than 10 million people are living with HCV, with high morbidity and mortality and most prevalent genotype of HCV was 3a [5]. HCV disease severity and its treatment response are greatly influenced by its nature [6]. Peg-interferon and ribavirin have been used with highest positive response rates for the treatment of genotypes 2 and 3 chronic HCV infection in developed countries [7–9]. It is ever challenging to characterize HCV heterogeneity and its treatment in developing countries due to different aspects including immunologic stress, HCV relapse rate, insignificant epidemiological studies, therapeutic cost, and patient's access to treatment of disease complications over time [10]. This comparative study is therefore designed to define the influence of genetic variation on the structural architecture of HCV NS3 GT3a, that is, which was previously studied extensively by cloning of NS3-GT3a protease from local population [11].

NS3 protein has two separate independent functional domains. A serine protease domain localized to the 181 residues of N-terminal, which acts to cleave HCV polyprotein and RNA/DNA helicase composed of 456 amino acids in C-terminal. Helicase portion unwinds duplex RNAs formed during the viral RNA replication in a 3′-5′ direction [12]. Inquisitively, the HCV helicase unwinds DNA more efficiently than RNA [13] even though the absence of any DNA intermediates in the HCV virulence cycle.

Several crystal structures of HCV NS3 including PDB IDs 3O8B [14], 1CU1 [15], 2ZJO [16], 1A1V [17], 1HEI [18], 3KQN [19], 2F55 [20], and 8OHM [21] and mechanistic studies [12, 21–27] helped to predict the ATP derived energy coupled helicase activity but all of the studies exclusively focused on genotype 1a or 1b of HCV. However, no molecular architecture of full length HCV GT3a NS3 has been elucidated. Little is known about the impact of residue variations on the HCV GT3a NS3 activities.

*Objectives.* The NS3 was chosen for this study because it has a clear biological relevance and can be relatively modeled and characterized easily by using the predetermined crystal structures of HCV genotype 1 NS3. This study described the characterization of HCV NS3 GT3a in parallel to genotypes 1a and 1b NS3. The goal is to understand the structural architecture of HCV genome variability and to further define the conserved targets for antiviral agents.

## 2. Methods

The sequence of NS3 protein belonged to Pakistani strain of HCV genotype 3a previously reported (PK-NCVI/Pk3a NS3) with accession number (FJ839678) and NZL1 (NC_009824) was obtained from online database of Genbank maintained by NCBI. Only the sequence of PK-NCVI/Pk3a NS3 (FJ839678) was submitted to position-specific iterative BLAST [28] at NCBI to obtain homologous 3-dimensional (3D) structures of proteins deposited in the protein data bank (PDB). The further analysis was done based on highest sequence identity and smallest distance from PK-NCVI/Pk3a as potential templates with comparative modeling, including PDB Ids 3KQN [19] (X-ray structure of hepatitis C virus NS3 helicase in complex with ssRNA), 3O8B [14] (Crystal structure of HCV NS3 protein), and 1CU1 [15]. Sequences of crystal structures having PDB ids 3O8B [14], 1CU1 [15], 2ZJO [16], 1A1V [17], 1HEI [18], 2F55 [20], and 8OHM [21] were the other entries for NS3 protein in PDB and studied for protein-protein comparison. Multiple sequence analysis was carried out following an established protocol using ClustalW [29] and T-Coffee [30] along with rapid scanning and correction of multiple sequence alignments [31, 32]. The resulting multiple alignments were visualized and annotated using the sequence alignment protocol in CLC sequence viewer (CLCBIO, Aarhus, Denmark). The homology model for consensus sequence was produced using MODELLER [33, 34]. The different structural models were produced and clustered into groups by using NMRCLUST [35] based on the root mean square distance (RMSD) between the corresponding residues in their structures. Best model was selected using the results of NMRCLUST, ERRAT score [36], and MODELLER calculated energy score of the model, as criteria. All of the protein assessment reported optimized comparative model of PK-NCVI/Pk3a NS3. Molecular operating environment (MOE) software: chemical computing group's molecular operating environment (MOE) and discovery studio were used for further protein analysis [37].

## 3. Results

### 3.1. Phylogenetic Analysis and Structural Comparison of HCV NS3 Protein

**Figure 1:**
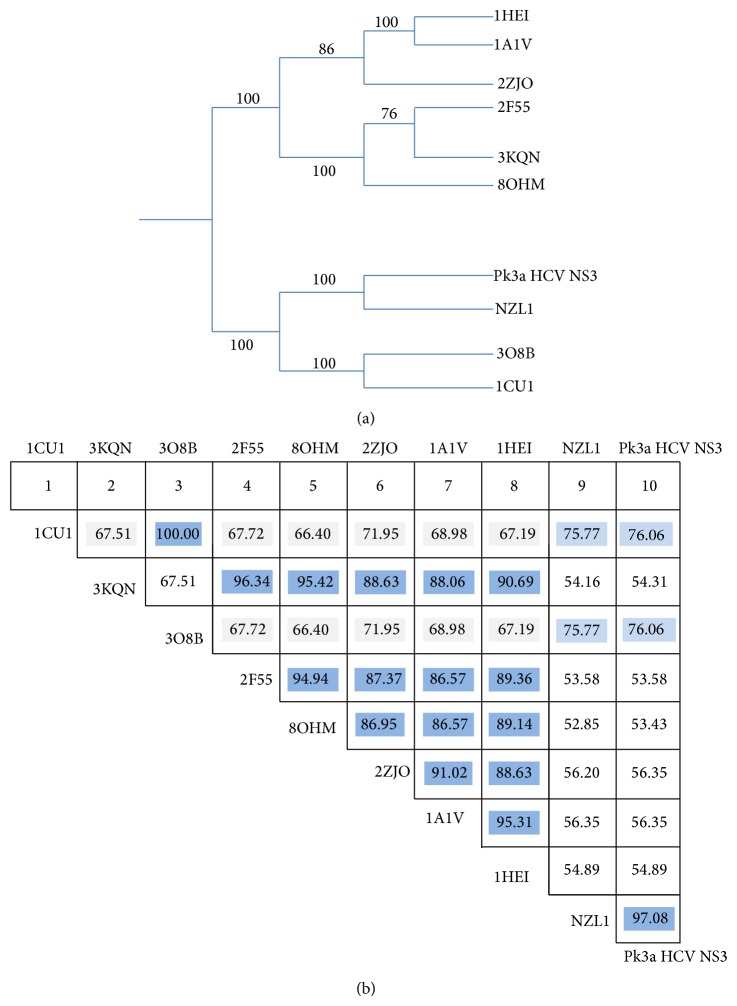
Sequence comparisons of the HCV NS3 genotypes. (a) Neighbour-joining tree based on phylogenetic analysis showing the relationships of HCV NS3 GT3a with GT1a/1b (b). Diagonal represents the comparison of percentage identities among crystal structure of HCV NS3 with Pk3a HCV NS3 and HCV NS3 of NZL1 strain. Dark colored values showed a higher percentage identity among cross matched sequence.

**Figure 2:**
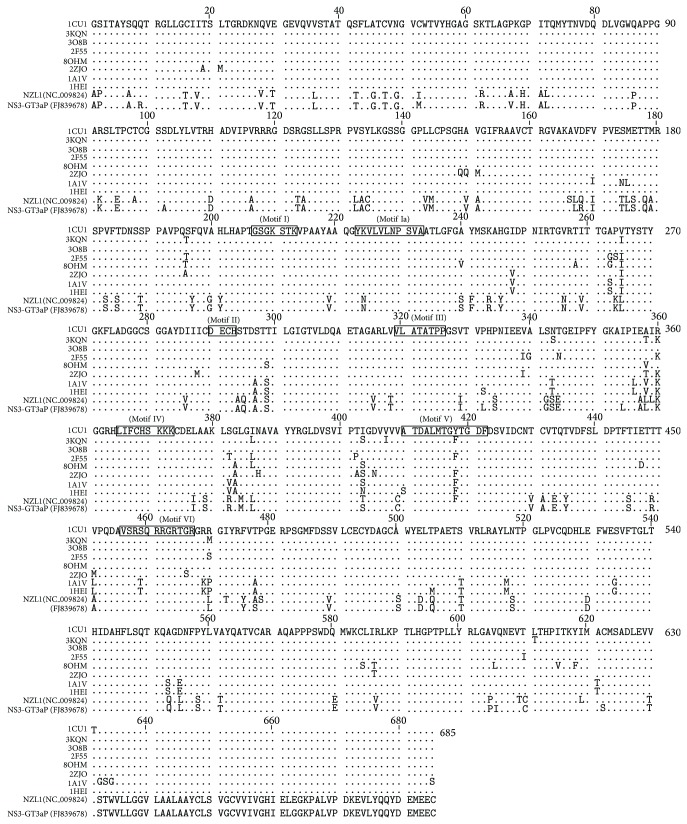
Multiple sequence alignment of different reported NS3 structures and target sequences of PK-NCVI/Pk3a NS3. Motifs I-VI are marked by boxes on the first sequence and labeled, respectively. The conserved sequences are shown as dots.

**Figure 3:**
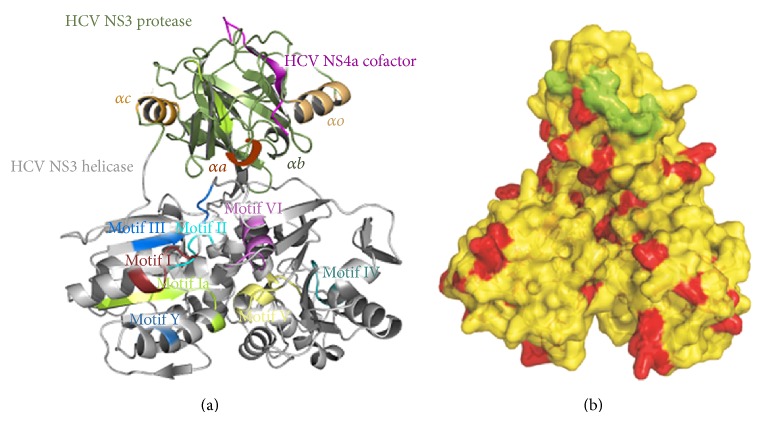
(a) Comparative structure of HCV NS3 GT3a (FJ839678). Different domains of protein along with its both protease and helicase sections. (b) Light yellow portion shows the conservations of residues while dark red portion shows the subsitutent residues in differnet crystal strucutres of HCV NS3 and NS3 GT3a.

We have done the pairwise alignment and phylogenetic analysis in order to identify percentage identity of reported NS3 GT3a (NZL1) with our target sequence PK-NCVI/Pk3a NS3 by comparative modeling. The target sequence and crystallographic analysis of PDB Id. 3O8B and 1CU1 shared the phylogenetic branch of target sequence as compared to other crystallographic studies (Figure 1(a)). Phylogenetic analysis was further confirmed by the multiple alignment percentage identity of 76.06% among their residues by using ClustalW and T-Coffee along with rapid scanning and correction of multiple sequence alignments (Figure 1(b)). The resulting multiple alignments were visualized and annotated using the sequence alignment protocol in CLC sequence viewer. It was found that most of the sequence variations were divided among all of the domains but N-terminal and middle portion of the reported NS3 protein were comparatively rich in variations (Figure 2). NS3-NS4A is composed of six subdomains (Figure 3(a)). The helicase, usually referred to as *α*-helical subdomain, consists of two structurally related *β*-*α*-*β* subdomains and a third subdomain of three short *β* strands and seven helices. The protease domain is folded into dual barrel fold, like other members of chymotrypsin serine protease family. The 13-residue NS4A proved to be a protease activation domain and is considered to be the sixth subdomain (Figure 3(a)). By using NMRCLUST, sixty models were produced and clustered into groups. The best structural model was selected using the results of NMRCLUST, ERRAT score, and MODELLER. Inspection of the final structural model indicates that some major mutations are present in the helicase and non-functional regions of PK-NCVI/Pk3a which presents some new non-covalent intermolecular (IM) interactions alongside of conserved sequence (Figure 3(b)). Major domains including motifs I (Walker motif: G207SGKSTK), Ia (Y*223*KVLVLNPSVA), II (Walker B: D*290*ECH), III (V*319*LATATPP), IV (L*365*IFCHSKKK), V (A*410*TDALMTGYTGDF), and VI (V*456*SRSQRRGRTGR) were conserved in the all HCV NS3 proteins of different genotypes. Structural architecture of these motives presented that those motifs make a border to ATP binding cleft and project some residues into the nucleic acid binding site. The residues of these motives transform the chemical energy derived from ATP hydrolysis into a mechanical force necessary for helicase movements for the disruption of ssRNA or RNA base pairs. Analogous IM interaction pattern of those domains was seen in all of the crystal studies and comparative model of reported NS3 except for mutations of Lys224Asn in motif Ia and Tyr418Phe in motif V (Supplementary Figure 1 available online at http://dx.doi.org/10.1155/2014/749254). Results implicated that mutation in motif 1a was strengthening its confirmation while motif V was loosely bound.

The role of most of the key conserved residues including Ala204, Thr324, Lys210 Ser231, Ser370, Thr411, Thr266, Tyr267, Met288, Thr269, Asp290, Glu291, Cys292, His293, Thr322, His369, Glu493, Tyr392, Trp501, Arg39, Val432, Phe438, Phe444, Thr450, Gln460, Arg461, Arg462, Gly463, Arg464, Thr465, Gly466, and Arg467 of HCV genotype 1a NS3, using site directed mutagenesis, has already been investigated. The comparison of these residues along with their interactions in modeled and template protein has been depicted in Table 1.

Some residues were seen to make different interaction pattern with their neighbors especially in the presence of ssRNA, ATP and solvent molecules. In the crystallographic studies of helicase interaction with ssRNA PDB Id, 3KQN Gln460 interacts with Thr324 and Arg464 but in the model of PK-NCVI/Pk3a and other studies without ATP and ssRNA, this interaction was seen with His293 and Arg464. Some amino acids like Glu291, Cys292, His293, and Thr324 were evaluated to be on surface and create a hydrogen bond with water used as solvent during crystallographic studies which was absent in the model of PK-NCVI/Pk3a. Some of the residues, which are not part of the functional regions of protein and nonconserved in PK-NCVI/Pk3a NS3, were actually involved in the structure stability. These residues are Arg9Gln, Leu332Pro, Leu354Ile, Ile605Val, and Ser622Cys [11]. The comparison of the IM interactions among nonconserved residues of PK-NCVI/Pk3a NS3 and of HCV NS3 of other genotypes is listed in Table 2.

**Figure 4:**
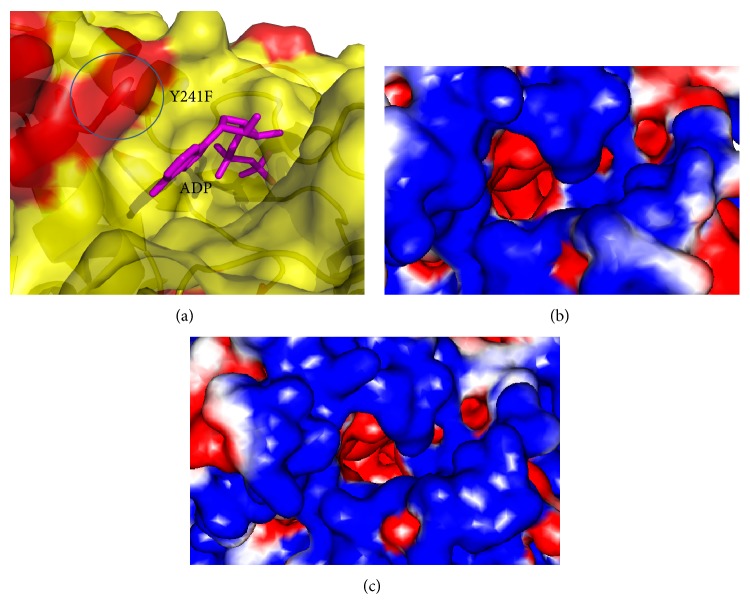
(a) ADP binding site in helicase portion of NS3 GT3a. (b) Comparison of electrostatic potentials of ADP binding site in NS3 GT3a and (c) template (PDB Id 3KQN).

The catalytic triad located in a cleft between two subdomains (or barrels), with His57 and Asp81 in the N-terminal subdomain and Ser139 in the C-terminal, has the similar behavior of IM interactions with their environment as shown in genotype 1a/1b. Along with other amino acids involved in making an active site of ATP molecules, Tyr241 and Thr419 sandwich the ADP adenine base (Figure 4). We found the mutation of Tyr241Phewhich can affect the ATP orientation and its effect on the helicase activity although the electrostatics of site produce changed environment. Main-chain nitrogen (from Gly207 to Thr212) and side-chain atoms (Lys210 and Thr212) were conserved and showed similar interactions in the model of PK-NCVI/Pk3a NS3. Previous mutagenesis and arginine methylation studies have already confirmed the importance of Lys210, Arg464, and Arg467 in the ATP binding site [38–41]. Gln460 and Ala323 along with three positively charged side chains of Lys210, Arg464, and Arg467 were conserved and showed the similar interactions with *γ*-phosphate of ATP.

### 3.2. Structural Comparison of HCV NS3 Protease Domain

Protease domain in HCV NS3 is covalently connected to helicase by a loop of residues including Pro182, Val183, Phe184, Thr185, and Asp186 which forms a solvent-exposed strand in all of the crystal structures of HCV NS3. Due to the flexible nature of this loop, different residues of protease and helicase domains get involved in making intradomain interaction. Mutations at position 183 and 185 with Ser in this loop of PK-NCVI/Pk3a NS3 presented almost conserved residues for protease-helicase interaction.

**Figure 5:**
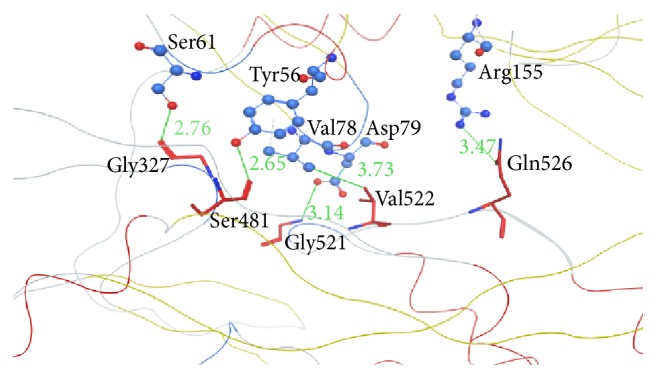
Interaction between HCV NS3 protease and helicase domains. Protease is showing in upper part of figure with atoms of residues as balls and helicase domain is the lower part of figure with atoms and bonds of involved residues shown as sticks. Secondary structure has been shown as lines.

The interaction site for the protease and helicase was also studied in different genotypes along with PK-NCVI/Pk3a NS3 (Figure 5). Interaction between helicase and protease normally involves Tyr56, Gly60, Ser61, Val78, and Asp79, Arg155 from the protease domain and Gly327, V329, Ser481, Pro482, Ser483, Gly484, Met485, Pro520, Gly521, Val522, Val524, and Gln526 from the side of helicase portion of NS3. These residues are conserved in all of the sequences except Val329 which is substituted with Ile329 in comparative model. The only conserved interactions seen in comparative model of NS3 for genotype 3a involved Tyr56, Ser61, Val78, and Asp79, Arg155, Gly327, Ser481, Gly521, Val522, and Gln526 (Figure 5) while rest of the IM contacts were not seen. This difference of interaction pattern between these two domains computationally confirms the orientation of protease domain with respect to helicase domain due to a flank motif connecting helicase and protease domain (Figure 5).

The residues of NS3 C-terminal 626–631 made an antiparallel *β*-sheet in extended conformation along the brink of protease involving His57, Lys136, Ser139, Arg155, Lys165, and Arg168 from protease domain and Asp625, Asp626, Glu628, Thr630, and Thr631 from the C-terminal in their interaction. Residues Lys136 and Ser139 were found in this interaction for the first time in our study and implicate some strong interaction of C-terminal with protease domain. Mutation of Val to Thr at position 630 showed conserved interaction pattern with protease domain. NS4a protein is involved in the completion of N-terminal protease of NS3 into barrel fold and the assembly of catalytic triad. Mutations of Ser7Ala, Gln9Arg, Gln28Val, and Glu30Thr were present at the borders of NS4a cofactor. Effects of these mutations were examined by running energy minimization protocol, which showed a different conformation for its folding. Similarly, mutations of Gln580Glu, Asp555Leu, Pro558Ser, Ala153Gln, Ala604Pro, Val256Asn, Thr449Arg, Thr430Val, Thr295Gln, Ser294Ala, Thr299Ser, and Ser297Ala were found to be present on either side of ssRNA pathway in helicase.

### 3.3. Structural Comparison of HCV NS3 Helicase Domain

**Figure 6:**
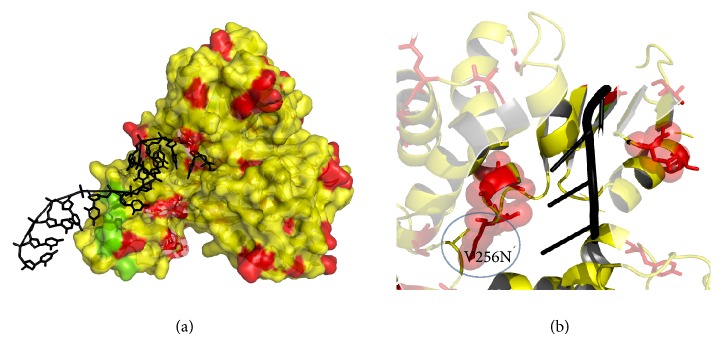
Two perspectives of ssDNA/ssRNA entry site in NS3. Black colored ssDNA was superimposed on the model using the template PDB Id 2F55. Red colored residues are the difference of residues seen in the multiple alignment of NS3GT3a with crystal studies.

**Figure 7:**
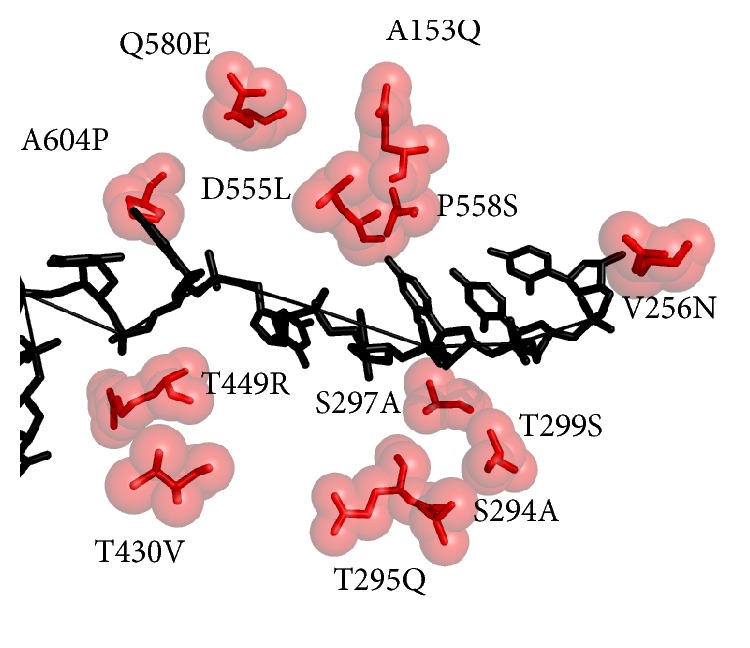
Superimposition of ssDNA from PDB Id 2F55 and NS3 GT3a model. Red sticks and spheres present the mutations seen in NS3 GT3a.

**Figure 8:**
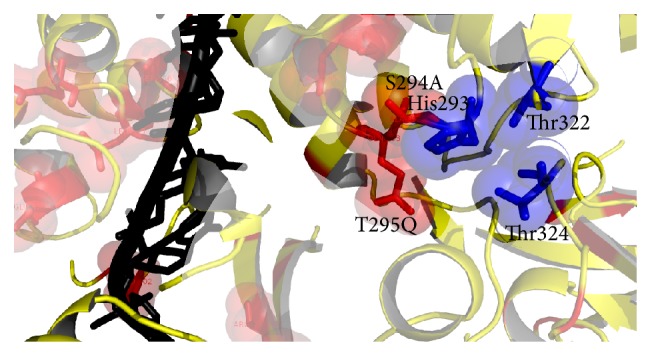
Residues Ser294Ala and Thr295Gln around coupling triad consist of His293, Thr322, and Thr324.

**Figure 9:**
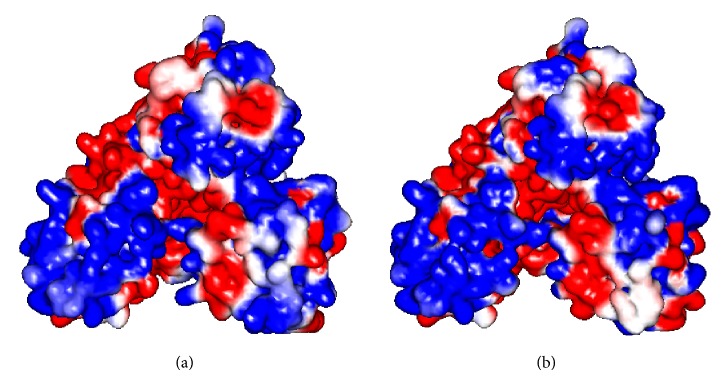
Electrostatic comparison of ssDNA/ssRNA entry site in (a) NS3 GT3a model and (b) crystal structure of HCV NS3 (PDB Id. 2F55).

**Figure 10:**
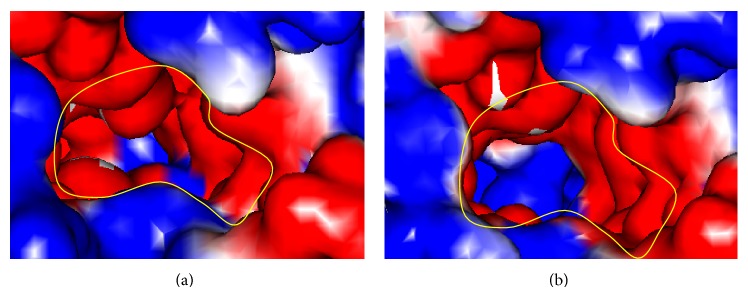
Comparison of internal space between the entry site of ssDNA/ssRNA in (a) NS3-GT3a model and (b) crystal structure of HCV NS3 without docked ssDNA/RNA (PDB Id. 3O8B).

The model of PK-NCVI/Pk3a was superimposed to the structure of NS3 (PDB Id. 2F55) and the residues involved in the helicase active site were characterized. The proposed ssDNA entry site in one of the dimer shown in PDB ID 2F55 can be seen in (Figure 6(a)). The entry site for ssDNA had a substitution of Val256Asn in homology model of PK-NCVI/Pk3a (Figure 6(b)). Mutations of Gln580Glu, Asp555Leu, Pro558Ser, Ala153Gln, Ala604Pro, Val256Asn, Thr449Arg, Thr430Val, Thr295Gln, Ser294Ala, Thr299Ser, and Ser297Ala were found to be present on either side of the entry of ssDNA (Figure 7). The NTP binding site was located at the periphery of the NTPase domain. The G*207*SGKST and the D*290*ECH sequences were forming lining of active site. One of the two loops connecting the RNA binding domain and NTPase had an invariant T*322*AT. His293, Thr322, and Thr324 are thought to function as a triad in coupling the helicase activity and NTP hydrolysis. We found that the neighbors of His293 (i.e., Ser294Ala and Thr295Gln) were non-conserved, which were affecting its flexibility to make hydrogen bonds, NTP hydrolysis, and the helicase activity (Figure 8). In the electrostatics computations for the ssDNA entry site, the probability of nonconserved residues in both genotypes presented a difference in the size of orifice for ssDNA/ssRNA entry (Figures 9 and 10). The probability for the small opening may result in a strong grip of helicase upon ssRNA which enhanced the helicase activity.

## 4. Discussion

The comparative structural modeling (CM) was used to predict the structure-function connection which remained undetectable at the sequences level. Several crystal structures of HCV NS3 and mechanistic studies helped to visualize helicase and protease activity but all of the studies exclusively focused on genotype 1a or 1b of HCV. Here, we used CM to investigate whether the PK-NCVI/Pk3a NS3 of Pakistani origin is employing some new key residues. The HCV NS3 protease along with 54 residues of NS4a protein is necessary to have a correct fold. In the absence of NS4a protein, NS3 protease is partially unfolded [42]. NS4a protein is involved in the completion of N-terminal protease of NS3 into barrel fold and the assembly of catalytic triad. Mutations of Ser7Ala, Gln9Arg, Gln28Val, and Glu30Thr were present at the borders of NS4a cofactor. The energy differences were also present and crystal structures of NS3 were at higher energy level as compared to the energy of PK-NCVI/Pk3a. Although, this procedure somewhat defines the folding pattern of NS4a in NS3 protease domain but further molecular dynamics simulations are needed which will provide some new insights about the folding of NS4a and its involvement in the stabilization of NS3 protease domain. Similarly, mutations of Gln580Glu, Asp555Leu, Pro558Ser, Ala153Gln, Ala604Pro, Val256Asn, Thr449Arg, Thr430Val, Thr295Gln, Ser294Ala, Thr299Ser, and Ser297Ala were found to be present on either side of ssRNA pathway in helicase. The effect of these mutations will be confirmed and validated by running QM-MM computational techniques in the further enhancement of these studies. Crystallographic studies (PDB Id: 2F55) have already confirmed the role of NS3 dimer formation and a long stretch of ssDNA passing through this dimer [20]. The NTP binding site was located at the periphery of the NTPase domain. The G*207*SGKST and the D*290*ECH sequences were forming lining of active site. The NTPase had an invariant T*322*AT. His293, Thr322, and Thr324 are thought to function as a triad in coupling the helicase activity and NTP hydrolysis. These nonconserved amino acids in the helicase portion may be involved in the strong binding of ssDNA/ssRNA, which may enhance the helicase efficiency of enzyme. The strong grip of helicase might be involved in the rate of ssDNA/ssRNA unwinding. Involvement of nonconserved residues is necessary for the determination of their roles in the rate of unwinding and determination of key residues for helicase inhibitors.

Analysis of the final model and the underlying alignment among PK-NCVI/Pk3a NS3 and reported genotypes of NS3 indicates that the mutations occur in the nonfunctional regions of PK-NCVI/Pk3a NS3 and almost conserved interactions between the residues have been seen. Hence, the substituted residues in PK-NCVI/Pk3a NS3 are not part of the functional regions of protein and are involved in the structure stability [11]. However, experimental techniques should be used to confirm the implications of structural differences and to investigate the congruency of our* in silico* results.

The IFN treatment response against the HCV genotype 3 is higher (~80%) in comparison to other strains (40%) [43]. However the new class of protease inhibitors seems to be less efficacious against genotype 3a, requiring higher concentration to achieve threshold level of inhibition as measured by qPCR of viral load. This may be due to the differences in the amino acids composition between genotype 1 versus genotype 3, as we have reported thus generating variable architecture and variable response. The biological relevance of the genotypic differences in NS3 has not been addressed in this study, but several intriguing ideas can be gleaned from the resultant data. These regions have less frequent mutations and possible substitutions are either positive in nature or have similar IM contact architecture. Although such ideas are highly speculative but they could be feasibly tested by measuring mutation rates in HCV replicon systems and could be measured in the presence and absence of ribavirin or other antiviral compounds. Altogether, this exercise reflected the interaction patterns of substituted residues along with implications for PK-NCVI/Pk3a NS3 in its role of disease profile and further studies with other genotypes should be carried out to define a complete map of IM noncovalent interactions responsible for the variable disease transmission, severity, and resistance to antiviral therapies.

## Supplementary Material

Figure 1. Multiple sequence alignment of different reported NS3 structures and target sequences of PK-NCVI/Pk3a NS3. Motifs I-VI are marked by boxes on the first sequence and labeled respectively. The conserved sequences are shown as dots.
Figure 2. Contact Maps of Lys224 (A) and Phe418 (B) with other residues in crystal studies of HCV NS3. Contact map of Asn224 (C) and Tyr418 (D) present in PK-NCVI/Pk3a NS3. Blue edges show the hydrogen bond, green show the hydrophobic interaction whereas red edges are showing the ionic bond formation between nodes.

## Figures and Tables

**Table 1:** The intermolecular interactions shared between NS3 of HCV genotypes 1 and 3.

Residue	Interactions shared among model and templates (bonding → atom of residue)
Ala204	HB → NZ of Lys210

Lys210	HB → O of Ala204
	HB → O of Pro205

Ser231	HB → OG1 of Thr235
	HB → O of Thr416

Thr266	Hb → OH of Tyr284

Tyr267	HB → O of Ser211

Ile288	HYD → CB of Val319

Asp290	HB → OG of Ser211
	HB → OH of Tyr270

His293	HB → OG1 of Thr322

His369	HB → OH of Tyr350

Tyr392	HB → OD2 of Asp375

Phe438	HYD → CB of Phe444
	HYD → CE2 of Phe531
	HYD → CE of Met623
	HYD → CZ2 of Trp532
	HYD → CD1 of Leu627

Phe444	HYD → CZ3 of Trp532
	HYD → CD1 of Leu560
	HYD → CG2 of Ile619
	HYD → CE of MET623

Gln460	HB → NE of Arg464

Arg461	HB → OD1 of Asp410
	HB → OD1 of Asp425

Arg462	HB → O of Val331
	HB → OE2 of Glu338

Gly463	HB → NH1 of Arg467

Arg464	HB → O of Tyr418
	HB → O of Gly420

Thr465	HB → O of Phe422
	HB → O of Asp423
	HB → OG1 of Thr465

Gly466	HB → ND2 of Asn335

Arg467	HB → O of Thr419

HB = hydrogen bond, Hb = hydrogen bond, CB = carbon atom with number, O = oxygen in hydroxyl group, OG = oxygen of amino acid with some number, that is, G, OG1 = oxygen with number, that is, G1, OH = oxygen with distinct number, that is, H, CE = carbon atom of amino acid with some number, that is, E, CE2 = carbon atom with some number, that is, E2, CD1 = carbon atom with some distinct number, that is, D1, NE = nitrogen atom with number, that is, E, NH1 = nitrogen atom with some number, that is, H1, HYD = hydrophobic bond.

**Table 2:** List of nonconserved residues in HCV NS3 genotype 3a structure in comparison with other HCV NS3 genotypes. Bold residues were seen shared among templates and models.

Position of the residue	Crystal structures		Model of NS3 GT3a	
	Residue	Interactions	Residue	Interactions
219	ALA	—	VAL	VAL225, ILE248, LEU265

224	LYS	ALA283, **ASP285**	ASN	**ASP285**

286	ILE	**LEU317, VAL319**	VAL	**LEU317, VAL319**

297	SER	GLU493	ALA	—

315	ALA	—	VAL	ILE285

354	ILE	ILE359, ILE366, **ILE426**	LEU	LEU341, **ILE426**, PHE349, LEU358

358	THR	—	LEU	LEU358

377	LEU	**LEU381**, VAL409	ILE	**LEU381**

384	LEU	—	MET	ILE345

407	ILE	—	VAL	ILE357, ILE364, LEU379

430	THR	**ARG461**	VAL	**ARG461**

433	THR	—	GLU	ARG479

445	THR	—	SER	SER439

477	THR	—	SER	ASN429, ARG458

489	SER	—	VAL	PHE557, VAL436, ILE446

500	ALA	LYS551	SER	ASP496

505	THR	—	GLN	THR509

510	SER	GLU533	THR	—

530	GLU	—	ASP	SER534

553	ALA	—	GLN	LYS583

561	VAL	MET581, TRP582, LEU602	THR	PHE557

605	VAL	VAL609	ILE	VAL609, LEU598

## Abbreviations

HCV: Hepatitis C virus
NS3: Nonstructural protein 3
kb: Kilo base
PK: Pakistan
3a: Genotype 3a
NS3: Nonstructural protein 3
DNA: Deoxy ribonucleic acid
ssRNA: Single stranded ribonucleic acid
QM/MM: Quantum mechanics/molecular mechanics
PDB: Protein data bank
IM: Intermolecular
ATP: Adenosine triphosphate
ADP: Adenine dinucleotide
NTPase: RNA nucleoside triphosphatases
NTP: Nucleoside triphosphate.
