# Supplementary material for: Comparison of Structural Architecture of HCV NS3 Genotype 1 versus Pakistani Genotype 3a
Source: Biomed Res Int. 2014 Oct 21;2014:749254. doi: 10.1155/2014/749254 (PMC4221965; doi:10.1155/2014/749254)
Supplement: Supplementary file 1 — Figure 1. Multiple sequence alignment of different reported NS3 structures and target sequences of PK-NCVI/Pk3a NS3. Motifs I-VI are marked by boxes on the first sequence and labeled respectively. The conserved sequences are shown as dots. Figure 2. Contact Maps of Lys224 (A) and Phe418 (B) with other residues in crystal studies of HCV NS3. Contact map of Asn224 (C) and Tyr418 (D) present in PK-NCVI/Pk3a NS3. Blue edges show the hydrogen bond, green show the hydrophobic interaction whereas red edges are showing the ionic bond formation between nodes. [file 749254.f1.doc]

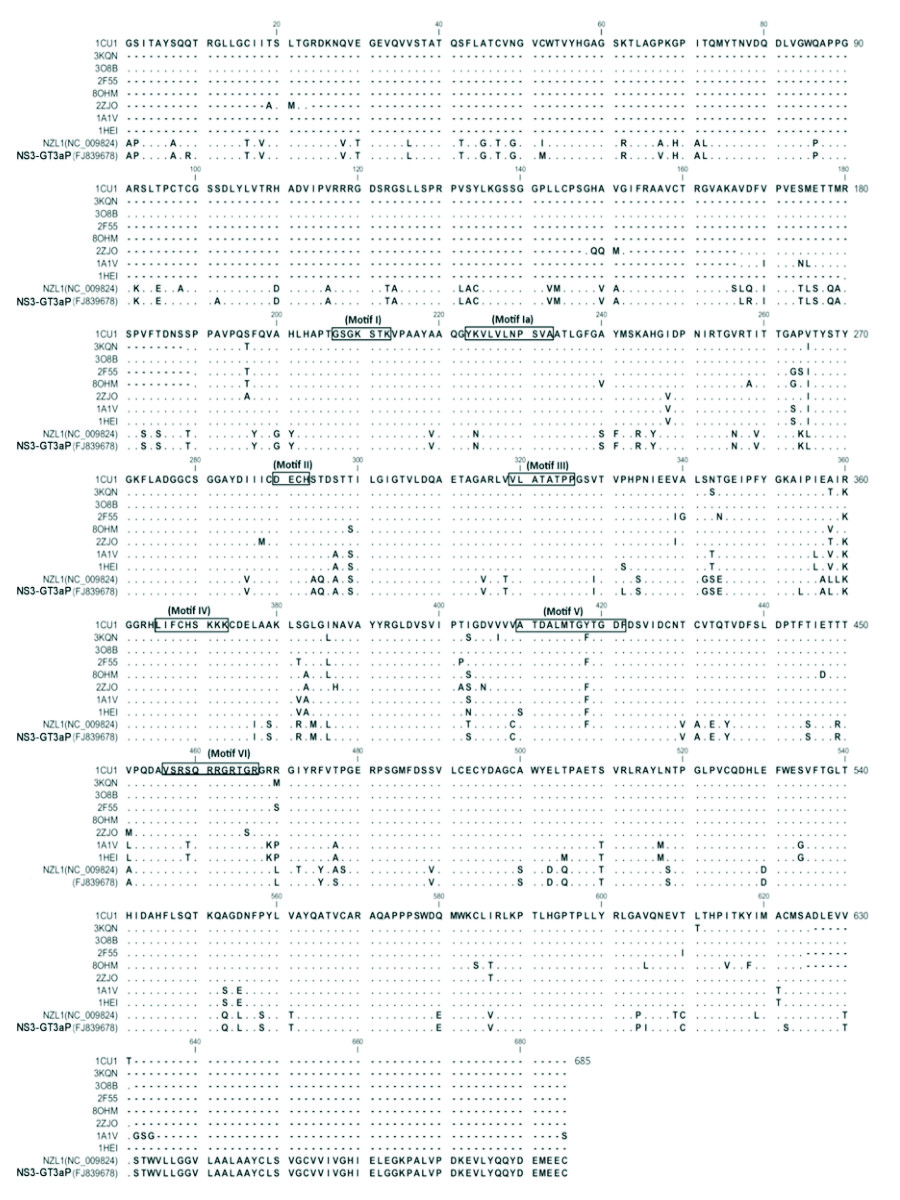


**Figure 1:** Multiple sequence alignment of different reported NS3 structures and target sequences of PK-NCVI/Pk3a NS3. Motifs I-VI are marked by boxes on the first sequence and labeled respectively. The conserved sequences are shown as dots.

**A B**

| 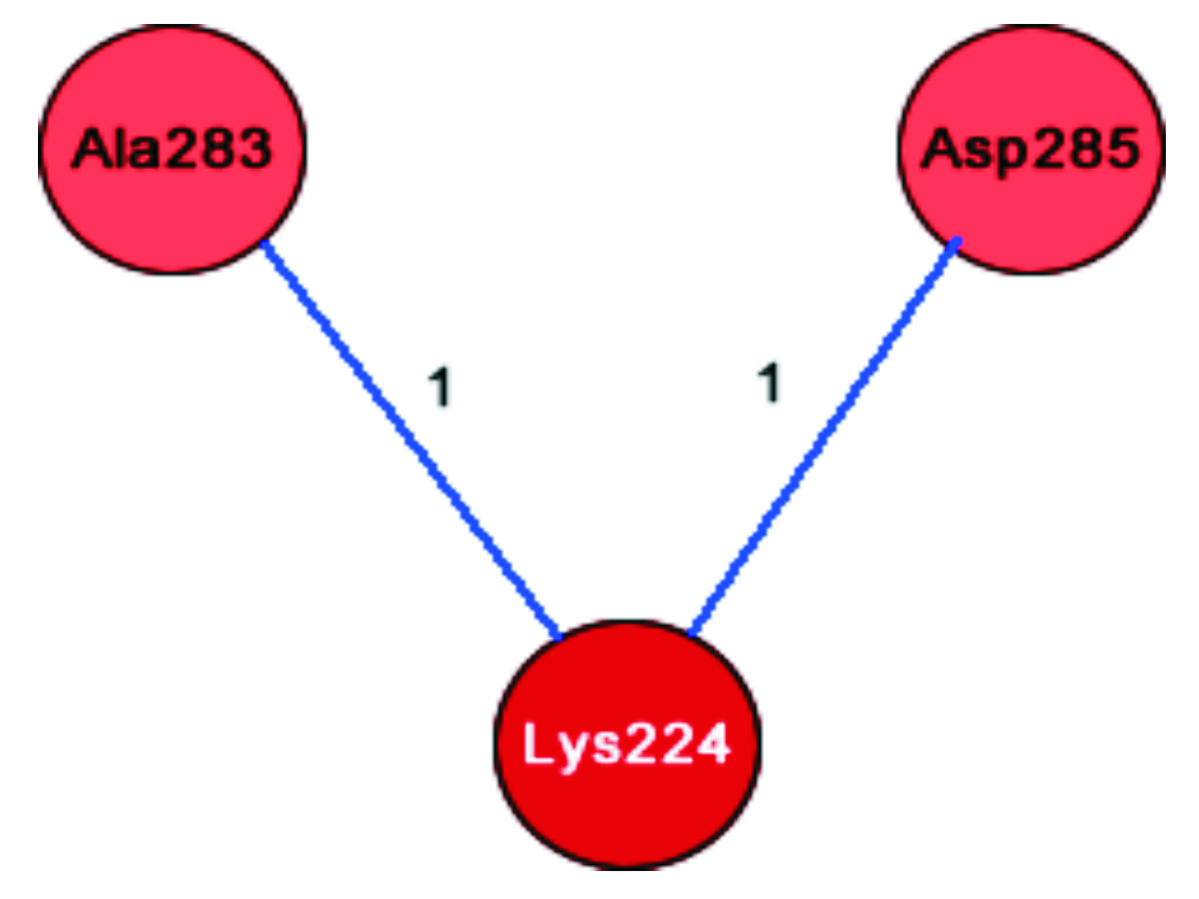 | 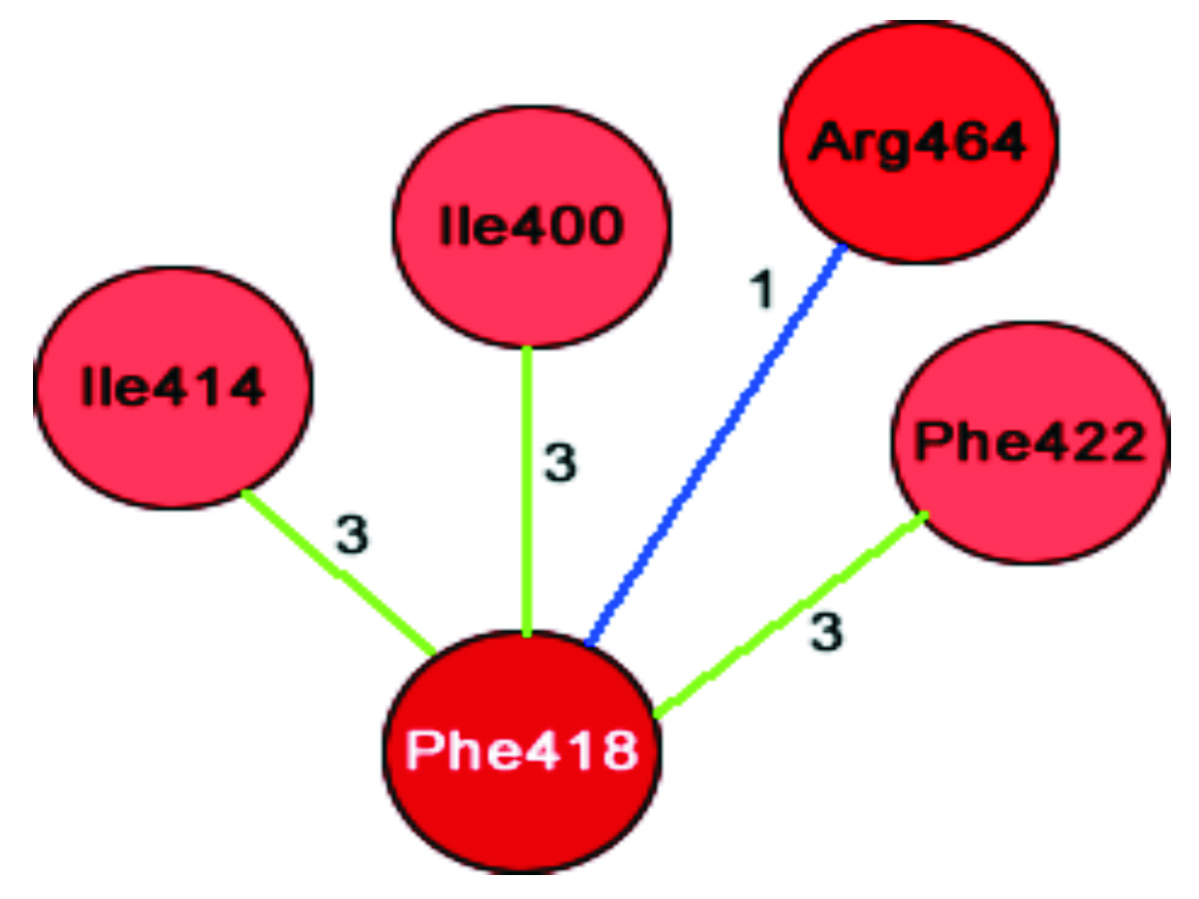 |
| --- | --- |
| **C** | **D** |
|  |  |
| 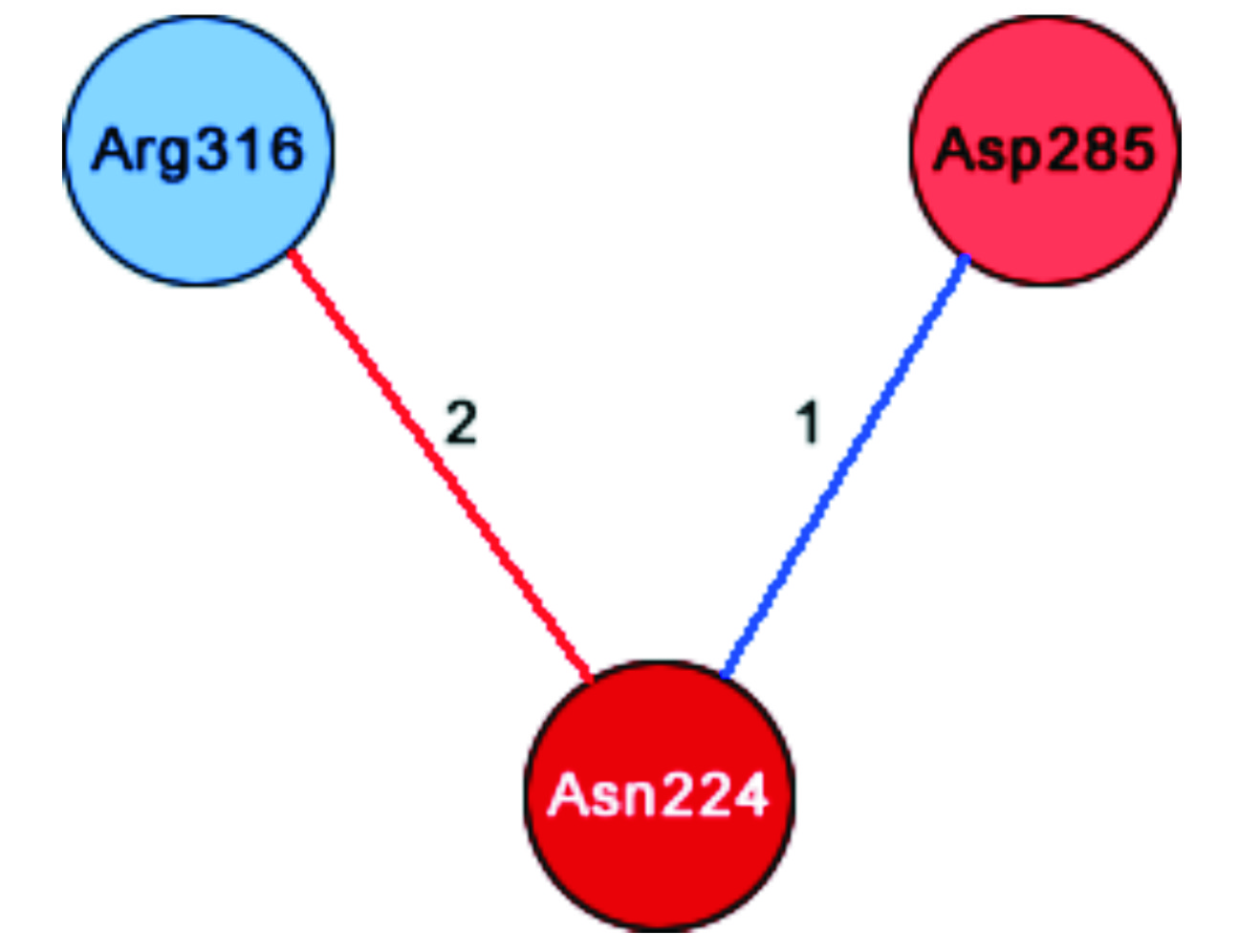 | 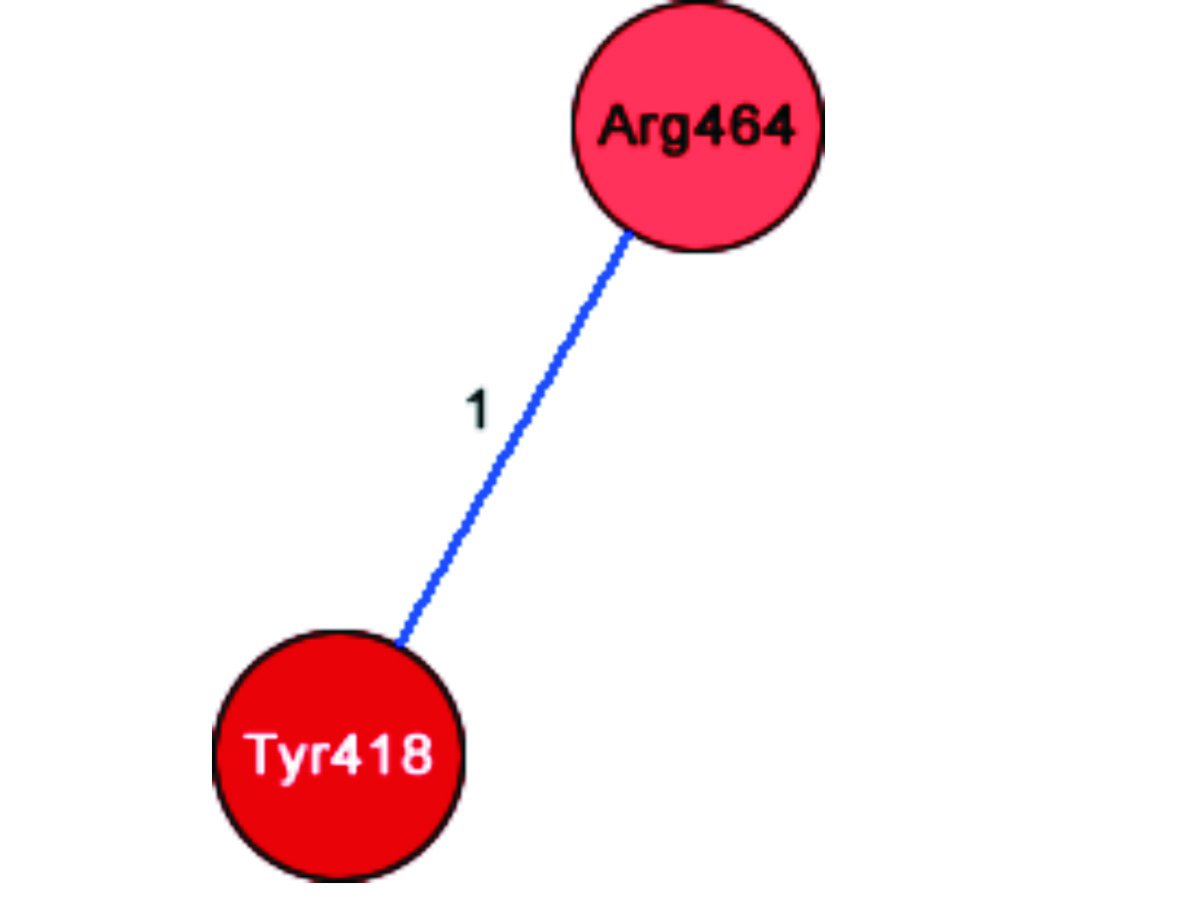 |

**Figure 2:** Contact Maps of Lys224 (A) and Phe418 (B) with other residues in crystal studies of HCV NS3. Contact map of Asn224 (C) and Tyr418 (D) present in PK-NCVI/Pk3a NS3. Blue edges show the hydrogen bond, green show the hydrophobic interaction whereas red edges are showing the ionic bond formation between nodes.
